# Surgical Resection of a Pneumothorax in an Adult Patient With a History of Wilson-Mikity Syndrome Diagnosed in Childhood

**DOI:** 10.7759/cureus.54641

**Published:** 2024-02-21

**Authors:** Ryusei Yoshino, Masaki Nakatsubo, Nanami Ujiie, Masahiro Kitada

**Affiliations:** 1 Thoracic Surgery and Breast Surgery, Asahikawa Medical University Hospital, Asahikawa, JPN

**Keywords:** surgery, chronic obstructive pulmonary disease, pneumothorax, chronic lung disease, wilson-mikity syndrome

## Abstract

Wilson-Mikity syndrome (WMS) is a rare condition characterized by various respiratory and pulmonary abnormalities in neonates and infants. However, the diagnosis is based on the findings of physiological tests, such as respiratory function tests. Reports describing the histopathological features of WMS are limited. The patient was a 22-year-old woman with a history of WMS. She had been on a ventilator for the first three months of life due to pulmonary hypertension after early delivery at 24 weeks of gestation and required oxygen therapy until three years of age. One month before presenting at our clinic, the patient experienced chest pain and respiratory distress, and a left spontaneous pneumothorax was diagnosed based on a chest X-ray examination. The pneumothorax improved after the insertion of a thoracic drain but recurred soon thereafter. A histopathological examination revealed emphysematous changes associated with WMS in the background lungs, consistent with brevity. No postoperative complications were observed. The thoracic drain was removed on the second day, and the patient was discharged on the eighth postoperative day. Postoperatively, the patient was started on inhaled medication and was carefully monitored every three months. The present case suggests that childhood interviews are very important for adult patients who develop pneumothorax and that early surgical treatment may be selected based on a detailed interview. Moreover, postoperative follow-up should be carefully performed in collaboration with respiratory medicine in patients with pneumothorax originating from chronic obstructive pulmonary diseases such as WMS.

## Introduction

Wilson-Mikity syndrome (WMS) is a rare condition characterized by various respiratory and pulmonary abnormalities in neonates and infants. WMS typically affects neonates who develop respiratory distress syndrome and are managed with ventilatory support; however, it is rarely mentioned, and the main type III chronic lung disease (CLD) is now treated as conventional WMS [1-3].

Reports on WMS are limited, and although CLD may develop in adulthood, both diagnoses are based on the results of physiological function tests, such as respiratory function tests. However, reports describing the actual pathological features are limited. In this case, WMS was diagnosed in the neonatal period, and a pneumothorax developed more than 20 years later. Histopathologically, the patient showed features of advanced chronic obstructive pulmonary disease (COPD) in early adulthood, and further disease progression was expected. Here, we report the progress of this case.

## Case presentation

A 22-year-old woman was diagnosed with a left spontaneous pneumothorax on chest radiography. A 12Fr aspiration kit was inserted; however, little improvement was noted, and the tube was replaced by a 20Fr thoracic drain. The patient was hospitalized for approximately 10 days. One week later, however, chest pain and dyspnea recurred, and chest radiography revealed a spontaneous left pneumothorax. A 20Fr thoracic drain was inserted, but no improvement was noted, and surgical resection was performed because of the early recurrence.

The patient’s medical history included WMS, Hashimoto’s disease, cerebral palsy, scoliosis, and reflux esophagitis. She was born at 24 weeks of gestation and had received ventilatory support for the first three months of life because of pulmonary hypertension. She required oxygen therapy until three years of age but had no respiratory disease, including pneumonia. There was no personal or family history of smoking. A physical examination revealed a height of 153 cm, a weight of 36 kg, and a body mass index of 15.4 kg/m^2^. Weak breathing sounds were observed in the left lung field. The heart sounds were clear, and no enlarged cervical, supraclavicular, or axillary lymph nodes were noted.

**Figure 1:**
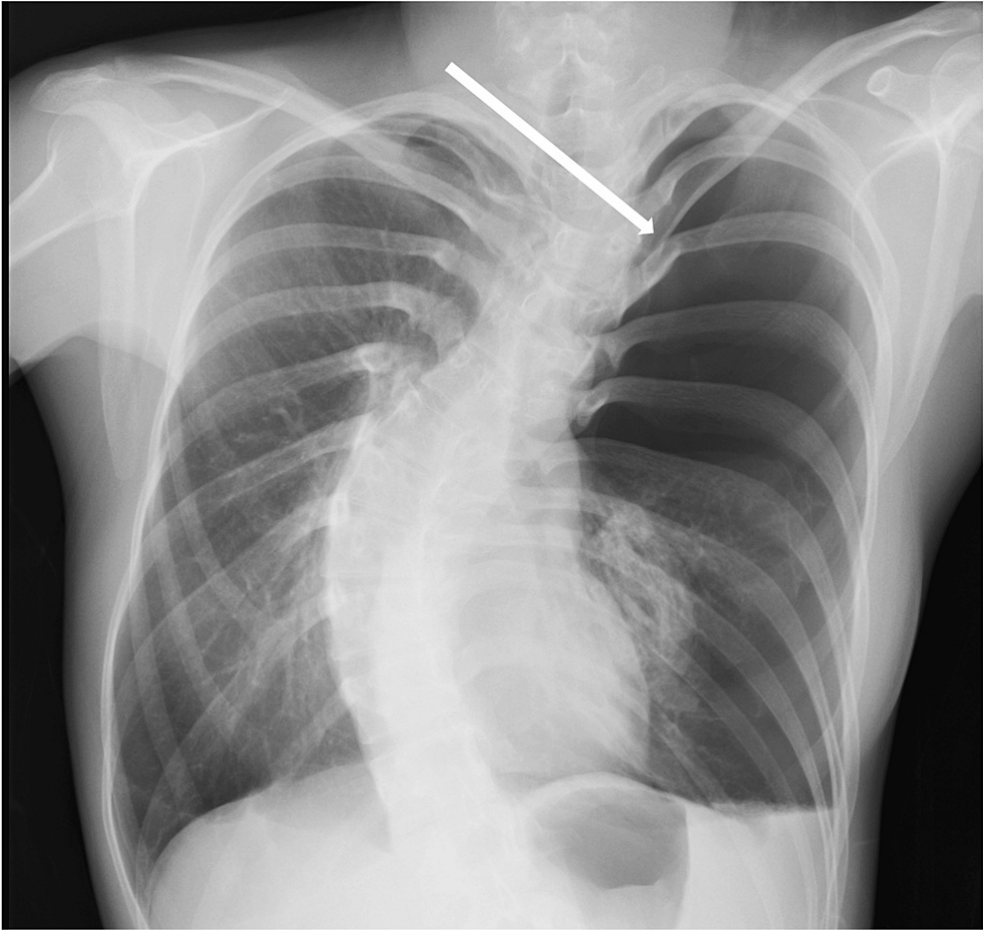
Chest radiograph (frontal view) The chest radiograph shows the collapse of the left lung.

**Figure 2:**
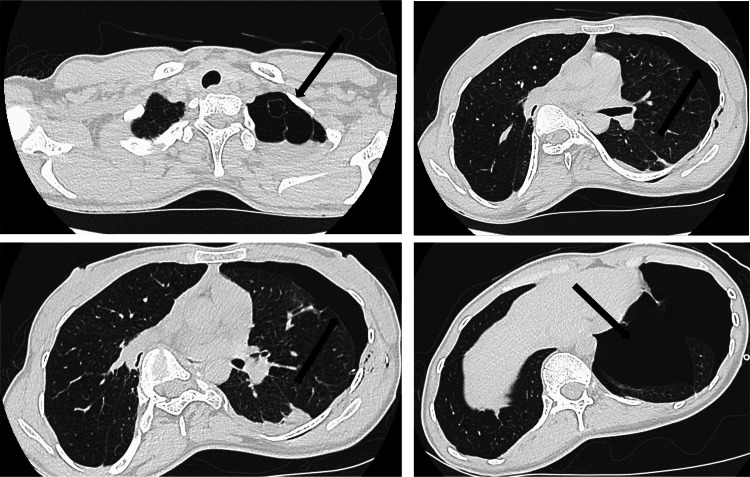
Chest computed tomography findings The chest computed tomography shows the collapse of the left lung and bilateral emphysematous changes.

Blood testing revealed no abnormal findings, including blood count, biochemistry, or coagulation. Electrocardiography revealed no other abnormal findings, and the patient was in sinus rhythm. No preoperative respiratory function tests were performed. Chest radiography revealed a collapsed left lung (Figure 1). A computed tomography scan of the chest also showed a collapsed left lung and emphysematous changes in the bilateral lungs (Figure 2). In addition, bilateral subcutaneous emphysema was observed. Based on these findings, the preoperative diagnosis was recurrent left spontaneous pneumothorax, for which a thoracoscopic partial left pulmonary cystectomy was performed.

**Figure 3:**
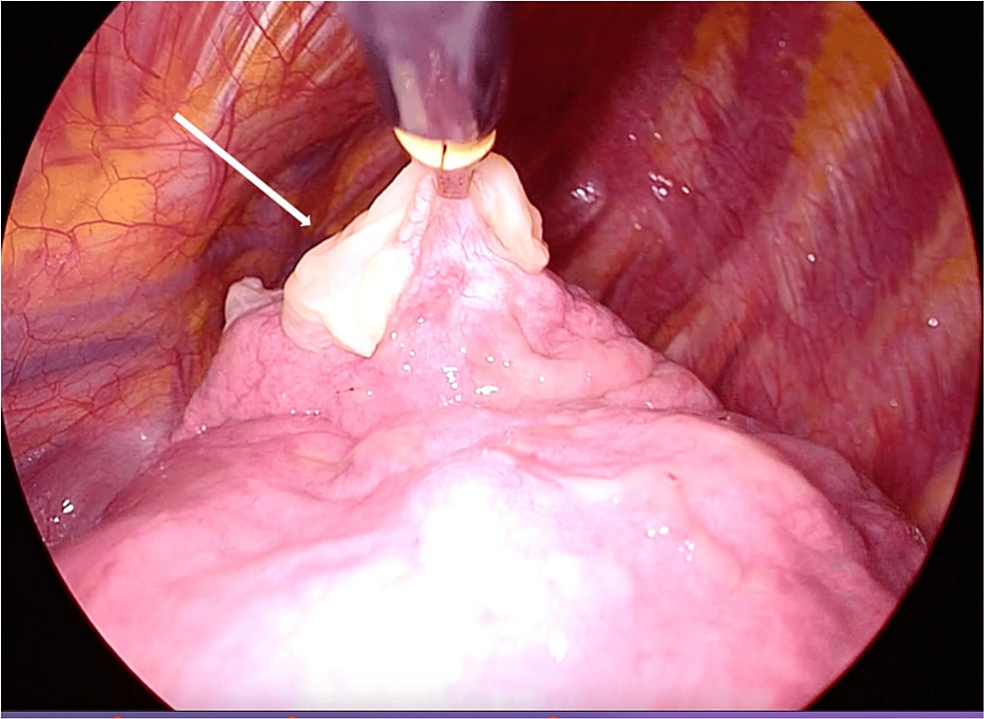
Intraoperative findings The intraoperative findings reveal grossly visible necrosis in the left pulmonary apex.

The patient underwent surgery under general anesthesia in the right lateral recumbent position. Three ports were used: one 10 mm above the mid-axillary line of the seventh intercostal space, another 7 mm above the anterior axillary line of the fifth intercostal space, and another 12 mm above the posterior axillary line of the fifth intercostal space. No adhesions were observed in the thoracic cavity. A cystic lesion was found at the apex of the lung, which was partially resected (Figure 3). The cyst had already been burned. A 24Fr thoracic drain was inserted, and the chest was closed. The operation time was 44 minutes, and the total blood loss was 10 mL.

**Figure 4:**
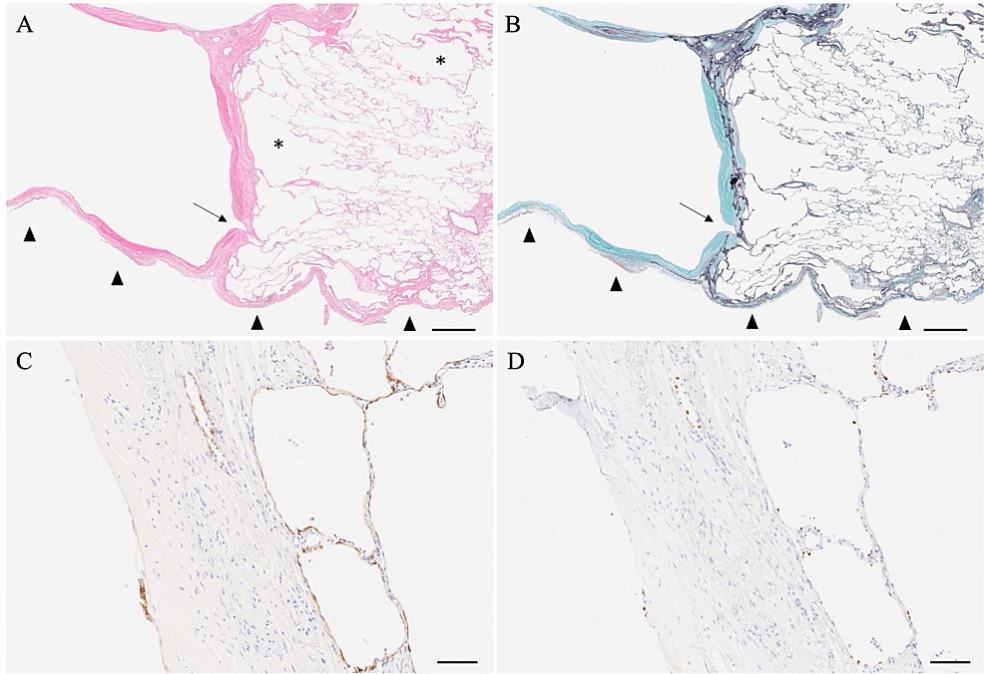
Pathological findings of the lung (A) and (B) Cysts are present within the pleura, with rare communication with the pulmonary alveoli (arrows). Emphysematous changes (*) are visible in the lung parenchyma. The arrowheads indicate pleural. (C) and (D) A few cells lining the cysts were identified and stained positive for Ber-EP4 and TTF-1. (Left side: cystic cavity; right side: alveoli.) (A) Hematoxylin and eosin stain; (B) Elastica-Masson stain; (C) Ber-EP4; (D) TTF-1. Scale bars: (A) and (B) 1 mm; (C) and (D) 100 μm.

A histopathological examination revealed that the cyst was composed of air retention in the pleura, and there was some traffic between the cyst and the alveoli. The cells lining the inner surface of the cyst were indistinct in many areas; in some areas, a single flat layer was observed. Immunohistochemical analyses were positive for both TTF-1 and BerEP4. Just below the cyst, collagen fibers filled the alveolar space along with elastic fibers, cholesterol clefts, and multinucleated giant cells. There was no evidence of endometriosis, estrogen receptor-positive cells, or lymphangioleiomyomatosis (Figure 4A-4D).

The patient had a good postoperative course. The thoracic drain was removed on the second postoperative day, and she was discharged on the eighth postoperative day. Postoperative respiratory function tests showed a vital capacity of 1,610 mL, a percentage vital capacity of 48.1%, forced expiratory volume in one second of 1,370 mL, and a percentage of forced expiratory volume in one second of 80.1%, indicating restricted ventilation failure. The patient had a history of WMS and was started on inhaled medication for COPD. The patient remains under careful follow-up every three months for possible progression of COPD, pulmonary hypertension, and heart failure due to cardiac overload.

## Discussion

WMS, first proposed by Wilson and Mikity in 1960, is a complication of respiratory distress syndrome, a lung disease that usually occurs in premature or underdeveloped neonates and is caused by a lack of pulmonary surfactant. WMS is characterized by the early development of cystic emphysema after ventilator management. However, this concept was recently abandoned [1-3]. Later, CLD was defined as persistent lung ventilation problems from the early neonatal period requiring oxygen therapy [1]. Type III CLD was diagnosed in most cases of foamy or cystic lungs in the neonatal period, as in WMS [4]. At that time, a relationship between intrauterine inflammation and CLD was suggested, and chorioamnionitis was reportedly a predisposing factor for type III CLD, the so-called WMS [3].

Childhood interviews are important for adult patients who develop pneumothorax, as their findings suggest the possibility of early surgical treatment options. The present patient had a history of WMS as a neonate and developed a pneumothorax as an adult. The patient was treated with multiple chest cavity drainages; however, surgical treatment was chosen because of the difficulty of a radical cure. Although we naturally checked her history and comorbidities as part of preoperative screening, it is difficult in actual clinical practice to focus on her history from the neonatal period to early childhood. Therefore, it is not possible to make a definite decision based on this case alone; however, focusing on a patient’s childhood medical history may be useful for predicting background lung disease and selecting early surgical treatment. Although WMS is thought to develop in the neonatal period and then progress to COPD [1], it is extremely difficult to perform a pathological evaluation during surgery; therefore, the histopathological findings of this case are very valuable.

Postoperative follow-up should be carefully performed in collaboration with the respiratory medicine department in patients with pneumothorax due to COPD, such as WMS. Although we found no reports of pneumothorax in patients with a history of WMS in the neonatal period, there have been case reports of obstructive lung disease at an early age [5]. The patient was suspected to have COPD based on histopathological findings of the lungs; it was considered possible that the COPD would progress in the long term, resulting in pulmonary hypertension and heart failure due to cardiac stress [6,7]. Therefore, postoperative respiratory function tests, a respiratory resistance test, and an inhalation test with a dilating agent were performed before the inhalant was started. In patients with COPD, such as WMS, careful postoperative follow-up is very important to monitor for possible long-term complications. Collaboration between surgeons and respiratory medicine specialists is essential.

Recent reports indicated that an intrauterine nutritional supply restriction due to maternal malnutrition may cause fetal growth retardation, resulting in pulmonary dysfunction, such as bronchopulmonary dysplasia, in the neonate. Conversely, maternal obesity during the perinatal period, fetal weight gain after birth, and overnutrition during childhood may cause COPD, such as asthma and wheezing [8]. Because the programming and mechanisms of such phenomena are unclear, future research is required. Smoking, air pollution, and abnormal intestinal microflora are known causes of CLD [8]. Therefore, it may be useful to ask patients who develop pneumothorax at a young age and have refractory disease about relevant environmental factors, including among their family members, in addition to their childhood medical history, and consider the possibility of CLD in the background lung. In addition, coronavirus infection has been suggested as a risk factor for pneumothorax [9]; with many humans now being exposed to coronaviruses, we can expect further identification of lung diseases in infants in the future.

## Conclusions

The current patient had a history of WMS as a neonate and developed a pneumothorax as an adult. The findings here suggest that childhood interviews are very important for adult patients with pneumothorax and that early surgical treatment may be selected based on detailed interview findings. In addition, postoperative follow-up of patients with pneumothorax who develop COPD, such as WMS, should be carefully performed in collaboration with the respiratory medicine department. In addition, support from other professions is essential for postoperative management, and rehabilitation medicine for postoperative rehabilitation and cardiology for preoperative and postoperative cardiac function must be involved in a comprehensive manner.
